# Genetic Polymorphisms in Exon 5 and Intron 5 and 7 of *AIRE* Are Associated with Rheumatoid Arthritis Risk in a Hungarian Population

**DOI:** 10.3390/biology13060439

**Published:** 2024-06-15

**Authors:** Bálint Bérczi, Nóra Nusser, Iván Péter, Balázs Németh, Ágota Kulisch, Zsuzsanna Kiss, Zoltán Gyöngyi

**Affiliations:** 1 Department of Public Health Medicine, Medical School, University of Pécs, Szigeti út 12, 7624 Pécs, Hungary; berczi.balint84@gmail.com (B.B.); balazs.nemeth@aok.pte.hu (B.N.); 2 Harkány Thermal Rehabilitation Centre, Zsigmondy Sétány 1, 7815 Harkány, Hungary; orvosigazgato@harkanykorhaz.hu (N.N.); foigazgato@harkanykorhaz.hu (I.P.); 3 St. Andrew Hospital for Rheumatology and Medicinal Spa of Hévíz, Dr. Schulhof Vilmos Sétány. 1, 8380 Hévíz, Hungary; agotakulisch@gmail.com (Á.K.); zsuzsanna92kiss@gmail.com (Z.K.)

**Keywords:** single-nucleotide polymorphism, central tolerance, rheumatoid arthritis, autoimmune disease

## Abstract

**Simple Summary:**

It is a fundamental question: what is the earliest commencement of autoimmune diseases? In recent years, one process deemed to be essential is central self-tolerance during the neonatal period, when the immune system selects and destroys autoimmune T-cells. This mechanism is genetically governed by the master gene *AIRE*. Genetic variations in this gene may cause a less efficient clearance of autoimmune T-cells, causing severe autoimmune diseases decades later. According to numerous research studies and our preliminary results, genetic polymorphisms in this master gene may be at the root of several autoimmune diseases. Although the interaction of genes and disease has been well studied in Asian populations, results are scarce in European populations. Our study is intended to analyse the association between genetic variations in this master gene and rheumatoid arthritis in subjects of Hungarian origin. We successfully identified an association between polymorphisms and the disease, reinforcing the notion that immunologic events during the neonatal period may contribute to autoimmune diseases. A further strength of our research is that in the future, because of its neonatal involvement, *AIRE* can be used as a risk biomarker in cases in which other regular markers cannot predict the disease risk.

**Abstract:**

Background: Rheumatoid arthritis (RA) is chronically persistent synovitis and systemic inflammation. Although multiple contributors are detected, only one is pivotal in the neonatal period: the negative selection of autoimmune naïve T-cells by the autoimmune regulator (AIRE) transcriptional factor. Methods: Single-nucleotide polymorphisms (SNPs) in the DNA-binding site of *AIRE* may determine its function and expression. We intended to analyse site-specific allelic polymorphisms in two exon (rs878081 and rs1055311) and three intron (rs1003853, rs2075876, and rs1003854) loci with an RA risk. Our analytical case-control study analysed 270 RA patients and 322 control subjects in five different genetic models using quantitative real-time PCR (qPCR) with TaqMan^®^ assays. Results: Statistically significant differences were found between the odds of allelic polymorphisms in the loci of rs878081, rs1003854, and rs1003853 among the controls and RA patients, and the disease activity seemed to be significantly associated with the genotypic subgroups of rs878081 and rs1055311. Our in silico analysis supported this, suggesting that allele-specific alterations in the binding affinity of transcriptional factor families might determine RA activity. Conclusion: Our findings highlight the involvement of neonatal self-tolerance in RA pathogenesis, providing novel insights into disease development and paving the way for an analysis of further site-specific genetic polymorphisms in *AIRE* to expand the intervention time for RA.

## 1. Introduction

Rheumatoid arthritis (RA) is a chronic autoimmune dysfunction associated with synovial inflammation in which consequent tissue destruction causes functional impairment and disability [1]. In medium- and high-income countries, the reported prevalence of RA is 0.5–1%, with an annual incidence of 5–50 cases per 100,000 people [1]. While the exact cause is not fully understood, it is widely accepted that in the central stage, the exo- and endogenous propagation of citrullination and autoreactive T-cells plays an essential role in the production of autoantibodies in lymph nodes, such as anti-cyclic citrullinated peptide (aCCP) antibodies, in the early stages of the disease [2,3]. The percolation of autoreactive CD4+ T-cells, plasma cells, and autoantibodies into the synovium initiates the later, destructive phase of RA, characterised by cytokine-driven synovitis and the formation of pannus by the proliferation of fibroblast-like synoviocytes (FLSs), leading to irreversible clinical manifestations [2,3,4,5,6].

RA was firstly found to be significantly associated with HLA-DRB1 alleles carrying a common amino acid motif, which is the human leukocyte antigen (HLA)-DRB shared epitope (HLA-DRB-SE); however, the dosage of the shared epitope allele is only associated with patients who present aCCP antibodies [7]. Subsequent high-throughput genome-wide association studies (GWASs) have highlighted the importance of non-HLA genes in the pathogenesis of RA [7,8,9]. As of the time of writing this article, three recent GWASs have identified 124 risk loci with RA, involving 69,825 patients and 330,798 adjusted controls [10,11,12].

The majority of these 124 loci are associated with general cellular and inflammatory processes. However, among these genes, only one, the autoimmune regulator (*AIRE*) is specifically linked to a neonatal immunologic event. Its crucial role is to suppress the autoimmune potential in neonates [13]. The *AIRE* gene’s coding sequence is located in the 21q22.3 chromosomal region with 14 exonic and 13 intronic regions. It encodes a 540-amino-acid transcription factor known as the autoimmune regulator (AIRE). This regulator is primarily produced in the thymic medulla and is localised in the nucleus of medullar thymoepithelial cells (mTECs). Its primary and crucial function is to eliminate autoimmune thymocytes from the repertoire by producing tissue-restricted antigens (TRAs) during promiscuous gene expression (pGE) [13,14,15,16,17]. The central role and expression of AIRE gradually disappear by the post-adolescent involution of the thymus, indicating its crucial role only in the neonatal period.

In the existing literature, the precise biological origin of RA has remained elusive. Thus, we aim to shed light on the early onset of the disease, suggesting neonatal self-tolerance as a potential premature contributor to RA. This suggests that the disease may start in the neonatal period.

AIRE comprises five functionally important structural domains from the N- to C-termini. These include the homogeneously staining region (HSR) or caspase-activation and recruitment domain (CARD), which are responsible for caspase activity during apoptosis and for controlling the connection with binding partners. The nuclear localisation signal (NLS) is recognised by specific importins. The SAND (Sp100 AIRE NucP41/75 and DEAF) domain contains a DNA-binding sequence for mTECs and has transcriptional characteristics that determine the production of TRAs. Additionally, two plant homeodomain (PHD1 and PHD2, respectively) proteins are isolated by one proline-rich region (PRR). These domains epigenetically interact with unmethylated histone H3 lysine 4 (H3K4) and DNA-dependent protein kinase (DNA-PK) and activate gene transcription, respectively [13,14,15,18,19]. Moreover, four LxxLL motifs are encased between the N- and C-termini, where their role is to interact with CREB-binding protein (CBP) and signal transducers and activators of transcription (STAT) partners [19].

Over 100 nonsense, frameshift, and missense mutations have been identified in AIRE protein coding sequences. These mutations lead to autoimmune polyendocrine syndrome type 1 (APS-1), also known as autoimmune polyendocrinopathy candidiasis ectodermal dystrophy (APECED) (OMIM #240300). APS-1 is characterised by multi-organ, generalised deterioration attributed to the failure to eliminate self-reactive T-cells during early immunologic development [19,20].

Research studies using K/BxN mouse models of RA have provided evidence supporting the role of central tolerance in the development of RA. This indicated that a low presentation of self-antigens may lead to the synovial percolation of autoreactive CD4+ type 1 T helper (Th) 1, Th2, Th17, and regulatory T cells (Treg) in the late, destructive stage of RA [5,21,22]. The association between negative selection and single-nucleotide polymorphisms (SNPs) in AIRE was first described by an in vivo study in which *AIRE*-655R (rs117557896) and *AIRE*-230Y (rs751032) were detected [23]. The study demonstrated that the haplotypes *AIRE*-655G and *AIRE*-230T dramatically reduced *AIRE* transcription, influenced negative selection, and, therefore, elevated the risk of autoimmunity [23]. Albeit the growing scientific interest in AIRE, only a small number of studies have associated autoimmune disease susceptibility to allelic polymorphisms in *AIRE*, including vitiligo [24], alopecia areata [25], Addison’s disease [26], systemic sclerosis [27], autoimmune thyroiditis [27], and melanoma [28]. Among most molecular epidemiological studies, RA has been associated with *AIRE* SNPs [29]. In order to comprehensively address the allelic polymorphisms in *AIRE*, associated with RA in Asian populations, we performed a meta-analysis with rs2075876 and rs760426 [30]. To date, only one study has investigated *AIRE* SNPs in a Spanish population, analysing allelic frequencies [31]. The SAND domain caught our attention due to its perceived importance in binding AIRE and its partners to mTEC DNA, as well as in protein–protein interactions that play a role in determining the negative selection of central tolerance through the transcriptional expression of TRAs [32]. Our study’s primary aim was to assess the relationship between *AIRE* and RA using five different genetic models (allelic, dominant, recessive, codominant heterozygous, codominant homozygous, and overdominant) in order to establish risk associations and associated clinical phenotypes.

## 2. Materials and Methods

### 2.1. Inclusion and Exclusion Criteria of Variations

We performed a multistep preliminary selection method to separate the variations of interest from copy number variations (CNVs), point mutations, insertions, and deletions (indels). UCSC Genome Browser (GRCh38 assembly) and Ensembl automatic annotation of the human genome sequence (GRCh38.p14) were used as human genome databases [33,34].

The inclusion criteria were set with the utmost thoroughness, requiring the global minor allele frequency (MAF) to be between the values of 0.01 and 0.5 and the variation to be present in at least one of the following databases: NCBI dbSNP, 1000 Genomes Project Phase III (including the HapMap project), NHLBI Trans-Omics for Precision Medicine (human only), NHLBI GO Exome Sequencing Project (human only), Genome Aggregation Database (human only), and Exome Aggregation Consortium (human only) [35,36,37,38,39]. Some variations presented global MAF between 0.01 and 0.5. Still, when we scrutinised the allele frequencies in subpopulations of European (EUR) and Utah residents with Northern and Western European ancestry (CEU), MAF was lower than 0.01, which raised the probability of detection failure in our study population. Therefore, we excluded those variations due to low detection probability.

### 2.2. Ethical Approval

Our analytical case-control study was conducted following the guidelines of the Declaration of Helsinki. Our protocol was also approved by the Secretary of the Medical Research Council, Scientific and Research Ethics Committee of Hungary (protocol numbers 11871-7/2018/EÜIG on 24 April 2018 and 57142-5/2022/EÜIG on 8 November 2022). Informed consent was obtained from all subjects.

### 2.3. Study Subjects

Individuals receiving outpatient or inpatient care at the Harkány Thermal Rehabilitation Centre and St. Andrew Hospital for Rheumatology of Hévíz formed the study population of RA patients and controls, and we successfully included a total of 592 individuals. Voluntary participants received our study description sheet and gave their informed consent. Whole blood samples were consecutively collected in 4 mL citrate tubes after patients’ admission and stored at −75 °C. The patients in the RA study group were required to be older than 18, sign a consent statement, and have clinically diagnosed RA, based on the American College of Rheumatology classification, to be included [40]. Patients with concomitant autoimmune pathology in their past medical history, such as Sjögren’s syndrome, psoriasis, and systemic sclerosis, were excluded from the further analysis to lower the probability of confounding effects of autoimmune diseases other than RA. We finally included 270 patients in the RA group. For the control study group, we required subjects to be older than 18, to sign a consent statement, to not have symptomatic RA, based on the American College of Rheumatology classification, and to not have any autoimmune manifestation recorded in their medical history. The mean age of RA symptomatological onset is 65 years; therefore, to minimise the probability of latent cases among controls, we included participants aged 75 or older [1]. Furthermore, levels of C-reactive protein (CRP) lower than 20 mg/dL and rheumatoid factor (Rf) lower than 25 IU/mL IgG were necessary for inclusion in the control group, and we successfully included 322 subjects [41].

### 2.4. Assessment of Clinical Parameters

Complete blood count, cholesterol profile, blood sugar profile, erythrocyte sedimentation rate (ESR), CRP, sodium, potassium, urea, creatinine, glutamate-oxaloacetate transaminase, glutamate-pyruvate transaminase, gamma-glutamyl transferase, alkaline phosphatase, lactate dehydrogenase, creatine kinase, protein albumin, magnesium, phosphorus, and serum bilirubin levels were thoroughly assessed. To ensure the seropositivity of patients with RA and the seronegativity of healthy controls, we determined levels of Rf, aCCP, and antinuclear antibodies [42,43]. Based on the aforementioned clinical parameters and diagnostic criteria, functional status was calculated for each subject with RA by disease activity score with 28-joint counts (DAS28) and functional independence measure (FIM) [44]. To determine ESR, the Westergren method was applied to measure C-reactive protein (CRP) and Rf (IgG). Konelab Arena 20XT Clinical Chemistry Analyzer (Thermo Fisher Scientific, Waltham, MA, USA) was used, following the manufacturer’s recommendations [45]. All of the abovementioned basic laboratory tests, including ESR and CRP in addition to Rf, were performed at the Harkány Thermal Rehabilitation Centre. Immunoscan CCPlus enzyme-linked immunosorbent assay kit (Svar Life Sciences, Malmö, Sweden) was used to determine aCCP levels at the Department of Immunology and Biotechnology, Medical School, University of Pécs, Hungary, according to the manufacturer’s protocols.

### 2.5. DNA Extraction

Genomic DNA of patients with RA and controls was isolated using 0.5 mL whole blood lysed with 1 mL DNAzol BD reagent (Thermo Fisher Scientific, Waltham, MA, USA) following the manufacturer’s instructions. The precipitation of genomic DNA (gDNA) was performed at room temperature with 0.4 mL isopropanol for 5 min, and then the sample was centrifuged. After the supernatant was removed, the pellet was first washed with 0.5 mL DNAzol BD reagent, subsequently followed by a second wash with 1 mL 75% ethanol. Dried genomic DNA was dissolved in 200 μL Tris-EDTA buffer and quantitatively measured using NanoDrop One (Thermo Fisher Scientific, Waltham, MA, USA). Per reaction, 20 ng gDNA was used for quantitative real-time PCR (qPCR).

### 2.6. Genotyping AIRE Variants

Purification of gDNA was subsequently followed by multiplex qPCR testing. Samples of patients with RA and controls were genotyped using TaqMan technology. To perform accurate discrimination, we used Minor Groove Binder (MGB) TaqMan^®^ Assays (Thermo Fisher Scientific, Waltham, MA, USA) in which MGB at the 3′ ends of the probe increased the melting temperature, therefore stabilising probe–target hybrids. MGB TaqMan probes are significantly shorter than traditional probes, enabling these probes to better and more accurately discriminate between highly homologous allele sequences. Assays use one primer pair, a VIC dye-labelled probe (530 nm yellow channel) that detects the Allele 1 sequence and an FAM dye-labelled probe (470 nm green channel) that detects the Allele 2 sequence. Samples with signals only in VIC or FAM channels are homozygous for the given sequence, while samples with signals in both channels are considered heterozygous for the analysed SNP site. Quality-checked and functionally tested Taqman assays were utilised for rs878081 (assay ID: C___2978265_10), rs1003854 (assay ID: C___9480542_10), rs2075876 (assay ID: C__15863141_20), rs1055311 (assay ID: C___9480540_20), and rs1003853 (assay ID: C___2978264_1_) (catalogue 4351379). As per our protocol, 9 μL template gDNA (20 ng) was mixed with 1 μL TaqMan^®^ Assay and 10 μL TaqMan Genotyping Master Mix (catalogue 4,371,357) (Thermo Fisher Scientific, Waltham, MA, USA) to a total volume of 20 μL. Fluorescence intensity was detected in both FAM and VIC channels in the Rotor-Gene 3000 thermal cycler (Corbett Life Science, Qiagen, Crawley, UK) with its software Rotor-Gene 6 v6.1 for which the protocol was as follows: 10 min at 95 °C for AmpliTaq Gold^®^ DNA Polymerase, UP enzyme activation, and 43 cycles of denaturation and synthesis (15 s at 95 °C for DNA denaturation, 1 min at 60 °C for annealing and extension). Assessment of allelic discrimination was conducted using Rotor-Gene 6000 v1.7 software (Corbett Life Science, Qiagen, Crawley, UK). Spectra of each sample were reviewed by visual inspection to confirm genotype assignment. The final call rate for genotyping was 100%.

### 2.7. Statistical Analyses

Clinical characteristics as continuous variables are represented as mean ± standard deviation (SD). Levene’s test was applied to analyse the equality of variances. Furthermore, the Shapiro–Wilk test was used to determine normality. Independent-sample t-tests and analysis of variance were used to compare groups for continuous variables. Non-normally distributed variables were compared by their medians, and Mann–Whitney U and Kruskal–Wallis H tests with two-tailed significance were used. To compare the significance of sex distributions among males and females, the one-sample Kolmogorov–Smirnov test was applied. Linkage disequilibrium (LD) was evaluated by Lewontin’s ‘D’, in which LD structure was constructed using Haploview software (version 4.2) [46,47]. Expected genotype frequencies were calculated based on Hardy–Weinberg equilibrium (HWE), and the difference with observed frequencies was statistically compared with non-parametric chi-square tests to see how well our observed frequencies fit the expected frequencies according to the HWE. To test the association between genotype and allele frequencies with RA, odds ratios (OR) with 95% confidence interval (CI) were calculated by binary logistics in which *p*-value less than 0.05 determined the statistical significance in allelic, codominant homozygous, codominant heterozygous, dominant, recessive, and overdominant genetic models. Bivariate correlation was applied with the Pearson correlation coefficient and two-tailed significance to determine the correlation between genotype frequencies and clinical characteristics. IBM SPSS software (version 28.0, IBM Company, Armonk, NY, USA) was used for statistical analyses.

### 2.8. Analysing the Effects of Variants on Regulatory Binding Motifs

To estimate the outcomes of allelic variants on *AIRE* expression in silico, we used HaploReg v4.2 databases [47]. Allele-specific affinity to transcription factor binding motifs was determined by using the logarithm of the odds (LOD) values of reference (ref) and alternative (alt) sequences [48]. When a positive result is provided by the subtraction of LOD (ref) from the LOD (alt) value for the alternative sequence, the predicted relative affinity is higher, suggesting that the alternate sequence binds the regulatory transcriptional factor with higher affinity than that of the reference sequence [48].

## 3. Results

### 3.1. Preliminarily Included Variations

In the UCSC Genome Browser and Ensembl automatic annotation system, our scope was restricted to the *AIRE* gene sequence (HGNC Symbol; Acc: HGNC:360), which resides in the 21q22.3 chromosomal region between 44,285,838 and 44,298,648 on the forward strand [33,34]. Without restrictions, we identified 23,023 variations. After adjusting the global MAF to 0.01–0.5, we detected 68 SNPs with at least one representation in NCBI dbSNP, 1000 Genomes Project Phase III (including the HapMap project), NHLBI Trans-Omics for Precision Medicine (human only), NHLBI GO Exome Sequencing Project (human only), Genome Aggregation Database (human only), and Exome Aggregation Consortium (human only) [35,36,37,38,39]. Following our exclusion criteria, we eliminated 24 SNPs with an MAF lower than 0.01 from the EUR and CEU subpopulations to avoid low chances of allelic discrimination; therefore, 44 variations were shortlisted in the 44,285,838–44,298,648 region.

**Figure 1:**
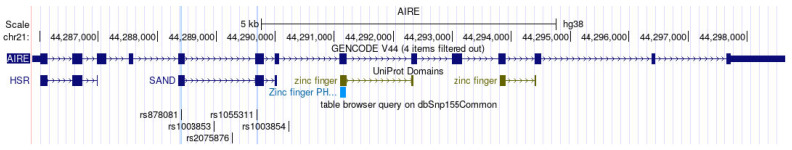
Exonic and intronic coding sequences in AIRE (GENCODE V44), domains of AIRE protein (UniProt domains), and positions of the five loci (table browser query on dbSnp155Common generated by UCSC Genome Browser on Human (GRCh38/hg38)). See https://genome.ucsc.edu/index.html (accessed on 25 March 2024) [33].

**Figure 2:**
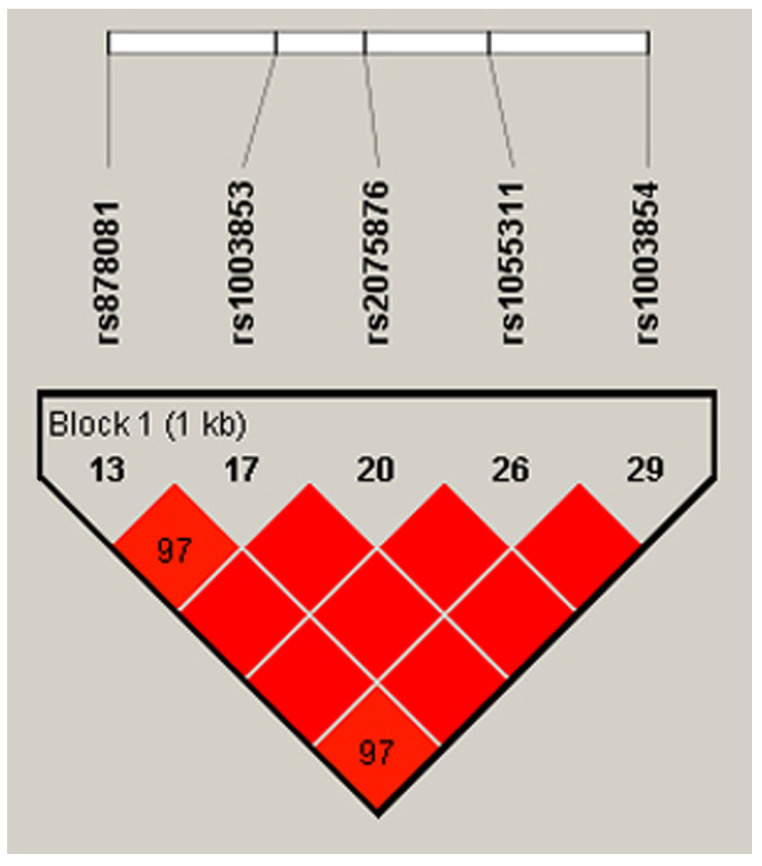
Positions of the five loci on the LD map and Lewontin’s ‘D’ values. The dark red colour indicates high LD, and blocks without numbers indicate the maximum LD value with ‘D’ = 1 (Species: Human; Assembly: GRCh38.p14; Region Lookup: 21:44285838-44298648). LD, linkage disequilibrium [46,47].

Out of the 14 exons in *AIRE*, Exon 5 and Exon 6 encode the SAND structural domain in particular, which is responsible for binding AIRE and its partners to mTECs’ DNA and further governing the pGE of TRAs by protein–protein interactions with CBP [32]. Mutations in SAND-encoding Exon 5 and Exon 6 and the splice site between them of Intron 5 cause autoimmune APECED [49,50,51,52]. In light of the importance of the SAND region in negative selection and autoimmunity, our scope of interest included the analysis of SNPs in Exons 5–6. Consequently, we chose the loci of rs878081 in Exon 5 and rs1055311 in Exon 6. Furthermore, we examined their strongly linked intron variants, including rs1003853, rs2075876 in Intron 5, and rs1003854 in Intron 7, respectively, to clarify the possible association of alleles and genotypes with RA and the clinical characteristics of patients. The positions of the two exon and three intron variations in *AIRE* and the resultant domains of AIRE are demonstrated in Figure 1. The five selected loci are on one strongly linked haplotype block with ‘D’ values ranging between 0.977 and 1.000. The linkage map is illustrated in Figure 2.

### 3.2. Characteristics of Study Populations

Our total population includes 592 individuals, 270 patients with RA, and 322 healthy controls. Among these, 100-100 participants were preliminarily investigated in our pilot study for allelic polymorphisms rs878081 and rs1003854 to see their tendencies of MAFs and to test our genotyping workflow and chances of detectability [50]. From then on, we formed two groups as follows: Group 1 was the total population with 270 RA patients and 322 controls for the rs1003853, rs2075876, and rs1055311 loci; and Group 2 had 170 RA patients and 222 controls, which was the subpopulation of Group 1 for rs878081 and rs1003854 because the results for 100 RA patients and 100 controls of rs878081 and rs1003854 from the total population in Group 1 had already been published. The characteristics of each study group can be seen in Table 1. The mean age of the RA patients was 65.47 ± 9.85 years and 65.53 ± 9.89 years, with 84.0% and 85.5% females in Groups 1 and 2, respectively. The distribution of females was significantly higher than that of males in the RA and control groups [1]. The participants in our total population were all born in Hungary. Their ESRs, CRPs, Rfs, and aCCPs were significantly higher at *p* < 0.001 in all of the RA groups with seropositivity values of 60.7% and 71.1%, respectively, and seronegativity values for control Groups 1 and 2 of 90.0% and 84.2%, respectively. Our RA groups seemed to be strongly positive with aCCP levels of 590.69 ± 947.96 and 596.27 ± 1003.71 U/mL and demonstrated a highly active disease (≥5.1) with DAS28 values calculated as 5.35 ± 1.20 and 5.44 ± 1.12 for Groups 1 and 2, respectively.

### 3.3. Results of HWE Analysis

The statistical significance levels of the difference between the expected and observed genotype frequencies in Group 1 for rs2075876, rs1055311, and rs1003853 among the RA patients were *p* = 0.916, *p* = 0.985, and *p* = 0.715, respectively. Those of the controls were *p* = 0.627, *p* = 0.899, and *p* = 0.503, respectively. In Group 2 for rs878081 and rs1003854, the statistical significance levels of the difference between the expected and observed genotype frequencies for each SNP among the RA patients were *p* = 0.991 and *p* = 0.995, respectively. Those of the controls were *p* = 0.870 and *p* = 0.658, respectively. Non-parametric chi-square tests demonstrated that the expected genotype frequencies fit well with the observed frequencies, following the HWE. Therefore, our study population is free from selection bias, non-random sampling, and the accumulation of relatives.

### 3.4. Allelic Polymorphisms in Exon 5 and Intron 5 and 7 Are Associated with RA Risk

To clarify significant associations between allelic and genotype frequencies and RA, allelic, codominant homozygous, codominant heterozygous, dominant, recessive, and overdominant genetic models were utilised for the 592 individuals and their 1184 alleles [51]. Among the five loci, the C-allele of SAND coding rs878081 on Exon 5 demonstrated firstly a significant association with RA risk under the allelic model (OR = 1.48, 95% CI 1.05–2.09, *p* = 0.023). The genotype frequency of CC homozygote RA patients was significantly higher and associated with RA risk under the recessive model in contrast with CT heterozygotes and TT homozygotes (OR = 1.64, 95% CI 1.09–2.48, *p* = 0.01). In the overdominant model, the CT genotype was negatively associated with RA risk, contrasting with the subpopulation of TT and CC homozygotes (OR = 0.64, 95% CI 0.42–0.97, *p* = 0.037). Supplementary Figures S1 and S2 show the allelic discrimination results of signals in VIC™ or FAM™ channels of rs878081 for the RA patients and controls.

Our study revealed a significant association between the intronic variation in rs1003853 in Intron 5 of *AIRE* and RA risk. Analysing the frequencies of 1184 alleles, we found that the C-allele was significantly more represented among the RA alleles than the alleles of the controls (OR = 1.33, 95% CI 1.01–1.74, *p* = 0.037). Interestingly, the CC homozygosity of rs1003853 was positively associated with RA risk by genotype frequency, with significantly higher odds for the RA patients than those for the controls in the recessive genetic model (OR = 1.618, 95% CI 1.16–2.25, *p*= 0.004). The overdominant model, however, suggested a negative association with RA risk (OR = 0.59, 95% CI 0.42–0.82, *p*= 0.002).

In the sequence of Intron 7 of *AIRE*, rs1003854 was the next locus where the odds of the T-allele were significantly higher for the RA alleles compared to those for the C-allele (OR = 1.52, 95% CI 1.08–2.12, *p* = 0.014). In the recessive model, TT homozygosity was significantly more frequent in the patients with RA than those with the TC and CC genotypes compared with the healthy controls (OR = 1.72, 95% CI 1.15–1.44, *p* = 0.008). In the overdominant model, heterozygotes were significantly overrepresented in the controls compared to in the RA cases (OR = 0.63, 95% CI 0.42–0.96, *p* = 0.034). The allele and genotype frequencies, as well as the results of our bivariate logistic regression associating rs878081, rs1003854, and rs1003853 with RA, are presented in Table 2. The association results of rs2075876 and rs1055311 with RA are published in Supplementary Tables S1 and S2.

### 3.5. Bivariate Correlation and Association of Clinical Parameters with Genotypic Subgroups of Different Genetic Models in Patients with RA and Control Subjects

Our study was intended to statistically analyse clinical parameters (ESR, CRP, Rf, aCCP, and DAS28) in the codominant homozygous, codominant heterozygous, dominant, recessive, and overdominant genetic models in subpopulations of RA patients and controls to provide possible evidence of differences between them in terms of clinical parameters and genotypes.

Among their clinical features, the clinical parameter ESR was able to characterise RA activity well by its significant positive correlation with DAS28 (*p* = 0.013) in the total RA population. Therefore, we decided to stratify the RA population based on codominant homozygous and heterozygous, dominant, recessive, and overdominant genetic models to see if there were further correlations and associations between genotypic subgroups and clinical parameters. Among the RA patients, statistically significant differences were found in the recessive model of rs878081, in which CC homozygotes presented significantly higher mean ESR levels (*p* = 0.027) compared with the TT + CT subpopulation. For the Pearson bivariate correlation, the ESR was shown to be significantly correlated with CC homozygotes (*p* = 0.023, r = 0.190); furthermore, with a binary logistic regression, a significant association (OR = 1.01, 95% CI 1.002–1.032, *p* = 0.026) was found. With 592 subjects, Group 1 demonstrated significantly higher aCCP levels among the 270 RA patients in the codominant heterozygous and dominant model of the rs1055311 locus (*p* = 0.028 and 0.044, respectively). Furthermore, our correlation analysis of the CT and CC genotypes revealed a significantly positive correlation of heterozygotes with significantly higher aCCP levels (r = 0.255, *p* = 0.036), suggesting a genotype-determined disease activity. The results of our subgroup analyses are demonstrated in Table 3 and Table 4.

### 3.6. Allele-Specific Affinity to Transcriptional Factor Binding Motifs

In the SAND domain, rs878081 on Exon 5 and rs1055311 on Exon 6 are synonymous polymorphisms that are not structural, but alterations in transcription may be associated with different alleles. To clarify the possible transcriptional consequences on alleles specifically, we performed an in silico analysis of the affinity of transcription factor binding motifs using HaploReg v4.2, in which we tested the four loci that seemed to be associated with RA risk and clinical parameters such as ESR and aCCP levels [48]. The database predicted two important motif-binding sites for nuclear factor-kappa B (NF-κB) and GATA transcription factors for the rs878081 and rs1003854 loci, respectively. GATA is a family of zinc-finger proteins with the ability to bind the consensus DNA sequence (T/A)GATA(A/G). The calculated LOD scores for affinity to regulatory motifs were +2 for the rs878081 T-allele and +1 for the rs1003854 C-allele, demonstrating a higher affinity of binding with the aforementioned transcriptional factors by alternate sequences of the rs878081 T-allele and rs1003854 C-allele, respectively. Furthermore, a higher affinity to bind to NF-κB was also predicted for the alternate rs1055311 T-allelic polymorphism with a +11.1 LOD score. Our results are demonstrated in Table 5.

## 4. Discussion

RA is a multifactorial autoimmune disease in which endo- and exogenous events contribute to the disease’s initiation [52]. Three of the latest GWASs have associated RA with specific loci posing a genetic risk, such as *AIRE*, which play a role in regulating immunological self-tolerance to ensure a healthy adaptive immune system. Postnatally, in a thymic environment, the thymocyte repertoire first undergoes an education against self-reactivity by central tolerance. Following this, peripheral organs further refine and eliminate potentially autoimmune T-cells through a process known as clonal deletion [53]. Central tolerance involves three checkpoints. The first is the β-selection, in which abnormal thymocytes with incorrectly assembled TCRs are removed [53]. The subsequent second checkpoint is the positive selection still in the cortex, where thymocytes are neglected if they are unable to recognise major histocompatibility complex (MHC) epitopes on cortical myoepithelial cells (ECs) [53]. Upon reaching the medulla, surviving thymocytes undergo negative selection at the final checkpoint [53]. At this stage, mTECs epigenetically express a diverse range of unique gene clusters, generating TRAs to mirror the immunologic “self” with the highest presentation from all parenchymal organs (such as the heart, stomach, bone, CNS, liver, and intestine) [53]. This phenomenon has been denoted as pGE, whereby AIRE binds to the DNA of mTECs by its SAND domain and regulates the expression of many downstream genes in clusters by binding their promoter or enhancer regions, ubiquitously and promiscuously synthesising TRAs as self-epitopes which are presented on MHC/HLA molecules of mTECs [53,54]. Incoming T-cells are immediately selected and clonally deleted if their TCRs recognise and interact with self-epitopes of mTECs because they seem to be autoimmune; moreover, medullar cells are also stopped due to apoptosis [53,54]. Binding AIRE to mTEC’s DNA is an essential step to establish a self-tolerant, mature T-cell repertoire. The DNA-binding SAND-domain coding region of *AIRE* plays an equally essential role in this process. Intronic and Exon 5 and 6 mutations in the SAND-domain coding sequence contribute to disrupted central tolerance, resulting in different forms of APECED [55,56,57,58].

The association of allelic polymorphisms and RA risk was initially identified in Asian populations, particularly in Japanese GWASs. In these study, it was found that rs2075876 and rs760426 were significantly associated with RA risk, with *p*-values of 5.1 × 10^−4^ and 2.0 × 10^−4^, respectively, among 471 SNPs [30,59,60,61,62,63]. Our study population accurately reflects the European population, and it is important to note that the allele frequencies and functions of the rs2075876 locus may differ in RA development compared to those in Asian populations. Possibly, that is why we could not find significant associations of rs2075876 with RA.

We believe the allelic polymorphisms in the DNA-binding SAND domain of this indispensable gene may lead to decreased AIRE expression, resulting in a diminished representation of TRAs during negative selection and allowing self-reactive thymocytes to evade elimination. The persistence of maturate autoreactive CD4^+^ T-cells promotes an environment conducive to RA, in which recruited autoimmune CD4^+^ T-cells in the synovium contribute to the development of inflammation by producing tumour necrosis factor-alpha, interleukins, and other proinflammatory cytokines, leading to tissue damage [64]. To associate the genetic exposure and disease endpoint part of our hypothesis, we intended to analyse five loci, for which the allelic polymorphism of rs878081 in Exon 5 of the SAND domain, rs1003853 in the Intron 5 splice site, and rs1003854 in the Intron 7 splice site were associated with RA risk.

To delve further into causality, we analysed the molecular consequences of our alleles of risk. The HaploReg database predicted that the two synonymous variations that reference the C-alleles of Exon 5 rs878081 and Exon 6 rs1055311 have lower affinities of binding to the DNA-binding motif of the NF-κB family, although rs1055311 was not associated with an RA risk but with a high level of aCCP and disease activity. NF-κB (RANK) signalling is essential in determining central and peripheral tolerance [65]. Numerous in vivo studies have demonstrated that RANK signaling significantly influences the expression of AIRE in both the thymus and extrathymic tissues. This is mediated through an evolutionarily conserved upstream NF-κB-responsive enhancer region. Studies on RANK-deficient mice have shown a decrease in thymic AIRE expression in mTECs, leading to the development of severe organ-specific autoimmunity. As a result, the NF-κB pathway plays a crucial role in regulating the expression of TRAs and the CCL19, CCL21, and CCR7 chemokines, which are responsible for facilitating the migration of thymocytes from the cortex to the medulla. [66,67]. NF-κB binding to the DNA of mTECs seemed to be crucial to *AIRE* expression. Thus, an allele-specific affinity for the transcriptional factor binding motif may provide different expression levels of *AIRE*. It is suggested that having two copies of low-affinity rs878081 C-alleles in homozygotes may genetically result in the lowest levels of AIRE and TRA expression, potentially leading to a higher number of surviving mature autoreactive CD4^+^ T-cells during development. This genetic variation may account for the significant association of the CC genotype with an increased risk of RA in the recessive genetic model. Within the RA population, it was observed that individuals with CC homozygosity exhibited significantly higher levels and a positive association with ESR, indicating disease activity. This finding supports the idea that genotype-dependent *AIRE* expression may not only determine one’s risk but also influence disease activity through clinical characteristics.

Furthermore, the T-allelic splice variant rs1003854 located in Intron 7 demonstrated a lower affinity to bind DNA-binding motifs of the GATA family, which is essential in the early and late stages of thymic and CD4^+^ thymocyte development from lymphoid progenitors [68]. In this transcriptional factor family, GATA3 governs the expression of TCR subunits, impacting β-selection and determining the commitment of CD4^+^ cell lineages in central tolerance [68]. As this allelic polymorphism is intronic, it is possible that it could influence alternative splicing through intron-mediated enhancement, thereby affecting AIRE expression [69]. Our research emphasizes the significance of allelic polymorphisms in negative selection, impacting the immunologic educator mTECs, the objects of selection, the naïve thymocytes, and their TCR development and β-selection.

The allelic and genotype frequencies of rs1055311 do not show a significant association with RA but are correlated with aCCP levels. The allelic polymorphism may be indirectly related to the disease through an intermediate variable, serum aCCP levels, without impacting the overall RA risk. Additionally, it may influence the survival of autoreactive T-cells that internally promote aCCP antibody production in an epistatic manner, characterising an intermediate phenotype of the disease. It is our view that the chosen allelic polymorphisms may impact both immunologic educators and educated sides.

Our research yields important tripartite clinical implications. Firstly, we have successfully associated the *AIRE* gene, crucial for neonatal self-tolerance with the onset of the disease. The presence of rs878081, rs1003854, and rs1003853 risk variants suggests a potential neonatal origin of RA, offering new insights into its development. Secondarily, we found that rs878081 and rs1055311 are genotypically associated with ESR and aCCP levels, respectively, in an RA population, providing further evidence that the lack of neonatal negative selection of self-reactive CD4^+^ T-cells against citrullinated peptides may lead to significantly higher disease activity and may explain the age-related aggregation of citrullination and autoantibodies [70]. Thirdly, *AIRE* variants are suitable for assessing the risk of RA, even in cases in which the patient is HLA-negative. Since the biological timing of the onset of RA starts around the neonatal period, there is longer time for intervention and monitoring. Our initial approach involves the study of AIRE, the principal regulator in central tolerance, and its DNA-binding coding region, given its pivotal role. It is crucial to note that TRA production relies on DNA binding. Once we achieve significant results, our next step will involve genetically mapping additional domain coding regions and analysing the genetics of the AIRE partners. We aim to broaden our focus beyond the medullary centre to include the corticomedullary and cortical regions, and ultimately the peripheral regions. Considering the intricate nature of neonatal self-tolerance, a stepwise analysis of the involved proteins and their coding regions is imperative.

Our limited ability to significantly associate allelic polymorphisms and clinical phenotypes with RA risk was mainly due to constraints in our sample size. Increasing the number of participants in our study would have significantly boosted our statistical power and provided greater certainty about the effect size. A further limitation is the scope of one domain coding region, SAND, whose results are not able to provide insight on AIRE. However, a genome-wide analysis of all allelic polymorphisms in the coding regions of all potential partners and their outcomes would not yield meaningful results without isolating and examining each player individually. Our findings could have been greatly corroborated with the analysis of the mRNA expression of *AIRE*, specifically associated with the five loci. To fully understand the link between RA and central tolerance, as well as the role of AIRE partners, it is necessary to obtain further genetic association results for the remaining 11 intronic and 12 exonic coding sequences.

## 5. Conclusions

In conclusion, our study’s findings are based on the analysis of the AIRE coding sequence. We have established significant associations between allelic polymorphisms and genotypes of the SAND domain coding Exon 5 and the Intron 5 and 7 splicing sites in relation to RA. We have also observed that the genetic subgroups of rs878081 and rs1055311 among the cases were significantly associated and correlated with the clinical parameters of the ESR and aCCP levels, respectively. These findings suggest a possible role of structural domain coding regions in AIRE in the development of RA, with implications for the potential origin of RA during neonatal self-tolerance. Moving forward, we aim to broaden our investigation to other domains with regulatory and epigenetic elements in order to enhance our understanding of gene function.

## Figures and Tables

**Table 1:** Clinical characteristics of study populations.

	Group 1		Group 2	
Characteristics	Patients with RA (n = 270)	Control Subjects (n = 322)	Patients with RA (n = 170)	Control Subjects (n = 222)
Age (years), mean ± SD	65.47 ± 9.85 ^a^	78.66 ± 5.86	65.53 ± 9.89 ^d^	77.53 ± 6.03
Sex				
Female, no. (%)	227 (84.0) ^b^	252 (78.2) ^c^	142 (83.5) ^e^	184 (82.8) ^f^
Male, no. (%)	43 (15.9)	70 (21.7)	28 (16.4)	38 (17.1)
ESR (mm/h), mean ± SD (normal range: 3–13 mm/h)	39.52 ± 25.21 ^a^	17.87 ± 12.81	39.09 ± 26.04 ^d^	19.02 ± 13.82
CRP (mg/dL), mean ± SD (normal range: 0–10 mg/dL)	29.79 ± 29.88 ^a^	7.10 ± 6.80	30.36 ± 30.34 ^d^	7.53 ± 8.03
Rf (IgG) (IU/mL), mean ± SD (normal range: 0–15 IU/mL)	118.80 ± 126.63 ^a^	13.49 ± 5.55	104.71 ± 113.31 ^d^	14.41 ± 13.75
aCCP (U/mL), mean ± SD (normal range: <20.0 U/mL; weak positivity: ≥20.0–39.0 U/mL; moderate positivity: ≥39.0–59.0 U/mL; strong positivity: ≥59.0 U/mL)	590.69 ± 947.96 ^a^	4.56 ± 2.29	596.27 ± 1003.71 ^d^	4.55 ± 1.66
Seropositive, no. (%)	164 (60.7)		121 (71.1)	
Seronegative, no. (%)	23 (8.5) ^a^	290 (90.0)	33 (19.4) ^d^	187 (84.2)
Without serology (%)	83 (30.8)	32 (10.0)	16 (9.5)	35 (15.8)
DAS28, mean ± SD (disease remission: <2.6; low: ≥2.6–3.1; moderate: ≥3.1–5.1; high: ≥5.1)	5.35 ± 1.20	-	5.44 ± 1.12	-

Group 1: total population; Group 2: subpopulation of the total; aCCP: anti-cyclic citrullinated peptide; CRP: C-reactive protein; DAS28: disease activity score with 28-joint counts; ESR: erythrocyte sedimentation rate; RA: rheumatoid arthritis; Rf: rheumatoid factor; SD: standard deviation; ^a^: *p* < 0.001 compared with control subjects in Group 1; ^b^: *p* < 0.001 compared with RA males in Group 1; ^c^: *p* < 0.001 compared with control males in Group 1; ^d^: *p* < 0.001 compared with control subjects in Group 2; ^e^: *p* < 0.001 compared with RA males in Group 2; ^f^: *p* < 0.01 compared with control males in Group 2.

**Table 2:** Allele and genotype frequencies, as well as association of RA with allelic polymorphisms rs878081, rs1003853, and rs1003854 in *AIRE*.

*AIRE* rs878081 (Exon 5)	RA n = 170 (%)	Control Subjects n = 222 (%)	OR (95% CI)	*p* ^a^
Alleles				
T	66 (19.4)	117 (26.4)		
C	274 (80.6)	327 (73.6)	1.48 (1.05–2.09)	0.023 *
Genotypes				
TT	7 (4.1)	13 (5.9)		
CT	53 (31.2)	92 (41.4)	1.07 (0.40–2.84)	0.892
CC	110 (64.7)	117 (52.7)	1.74 (0.67–4.53)	0.253
Dominant model				
TT	7 (4.1)	13 (5.9)		
CC + CT	163 (95.9)	209 (94.1)	1.44 (0.56–3.71)	0.441
Recessive model				
CT + TT	60 (35.3)	105 (47.3)		
CC	110 (64.7)	117 (52.7)	1.64 (1.09–2.48)	0.017 *
Overdominant model				
CC + TT	117 (68.8)	130 (58.6)		
CT	53 (31.2)	92 (41.4)	0.64 (0.42–0.97)	0.037 *
*AIRE* rs1003853 (Intron 5)	RA n = 270 (%)	Control subjects n = 322 (%)	OR (95% CI)	*p* ^a^
Alleles				
T	116 (21.5)	172 (26.7)		
C	424 (78.5)	472 (73.3)	1.33 (1.01–1.74)	0.037 *
Genotypes				
TT	15 (5.6)	16 (5.0)		
CT	85 (31.5)	141 (43.8)	0.64 (0.30–1.36)	0.251
CC	170 (63.0)	165 (51.2)	1.09 (0.52–2.29)	0.802
Dominant model				
TT	15 (5.6)	16 (5.0)		
CC + CT	255 (94.4)	306 (95.0)	0.88 (0.43–1.83)	0.750
Recessive model				
CT + TT	100 (37.0)	157 (48.8)		
CC	170 (63.0)	165 (51.2)	1.618 (1.16–2.25)	0.004 *
Overdominant model				
CC + TT	185 (68.5)	181 (56.2)		
CT	85 (31.5)	141 (43.8)	0.59 (0.42–0.82)	0.002 *
*AIRE* rs1003854 (Intron 7)	RA n = 170 (%)	Control subjects n = 222 (%)	OR (95% CI)	*p* ^a^
Alleles				
C	69 (20.3)	124 (27.9)		
T	271 (79.7)	320 (72.1)	1.52 (1.08–2.12)	0.014 *
Genotypes				
CC	6 (3.5)	13 (5.9)		
TC	57 (33.5)	99 (44.6)	1.34 (0.48–3.69)	0.567
TT	106 (62.4)	109 (41.1)	2.10 (0.77–5.74)	0.146
Dominant model				
CC	6 (3.5)	14 (6.3)		
TC + TT	164 (96.5)	208 (93.7)	1.84 (0.69–4.89)	0.222
Recessive model				
TC + CC	63 (37.1)	112 (50.5)		
TT	107 (62.9)	110 (49.5)	1.72 (1.15–1.44)	0.008 *
Overdominant model				
CC + TT	113 (66.5)	124 (55.9)		
TC	57 (33.5)	98 (44.1)	0.63 (0.42–0.96)	0.034 *

CI: confidence interval; OR: odds ratio; RA: rheumatoid arthritis. *p* ^a^: significance of binary logistic regression; * *p* < 0.05.

**Table 3:** Correlation and association of ESR levels with genotype subgroups in five different genetic models of rs878081 in *AIRE*.

*AIRE* rs878081 (Exon 5)	Patients with RA						Control Subjects					
	ESR	*p* ^a^	Pearson Correlation	*p* ^b^	OR (95% CI)	*p* ^c^	ESR	*p* ^d^	Pearson Correlation	*p* ^e^	OR (95% CI)	*p* ^f^
Genotype												
TT	24.25 ± 13.50						16.69 ± 13.11					
CT	33.16 ± 23.37	0.599	0.10	0.458	1.02 (0.96–1.08)	0.454	21.73 ± 14.96	0.147	0.11	0.255	1.02 (0.97–1.08)	0.259
CC	42.77 ± 27.11	0.196	0.13	0.179	1.03 (0.98–1.09)	0.196	17.33 ± 12.79	0.708	0.01	0.86	1.004 (0.95–1.05)	0.863
Dominant												
TT	24.25 ± 13.50						16.69 ± 13.11					
CC + CT	39.52 ± 26.22	0.295	0.09	0.24	1.03 (0.97–1.09)	0.262	19.18 ± 13.88	0.391	0.04	0.530	1.01 (0.96–1.06)	0.530
Recessive												
TT + CT	32.46 ± 22.79						21.04 ± 14.76					
CC	42.77 ± 27.11	0.027 *	0.19	0.023 *	1.01 (1.002–1.03)	0.026 *	17.33 ± 12.79	0.063	−0.13	0.054	0.98 (0.96–1.001)	0.980
Overdominant												
TT + CC	42.00 ± 26.90						17.26 ± 12.77					
CT	33.16 ± 23.37	0.059	−0.16	0.056	0.98 (0.97–1.001)	0.059	21.73 ± 14.96	0.020 *	0.15	0.023 *	1.02 (1.003–1.04)	0.025 *

ESR: erythrocyte sedimentation rate; OR: odds ratio; RA: rheumatoid arthritis; SD: standard deviation; ESR levels were expressed as mean ± SD; *p* ^a^: significance of differences in ESR levels in patients with RA; *p* ^b^: significance of Pearson correlation in genetic subgroups and ESR levels in patients with RA; *p* ^c^: significance of bivariate logistic regression of genetic subgroups and ESR levels in patients with RA; *p* ^d^: significance of differences in ESR levels in healthy controls; *p* ^e^: significance of Pearson correlation in genetic subgroups and ESR levels in healthy controls; *p* ^f^: significance of bivariate logistic regression of genetic subgroups and ESR levels in healthy controls; *: *p* < 0.05.

**Table 4:** Correlation of aCCP levels with genotype subgroups in five different genetic models of rs1055311 in *AIRE*.

*AIRE* rs1055311 (Exon 6)	Patients with RA				Control Subjects			
	aCCP	*p* ^a^	Pearson Correlation	*p* ^b^	aCCP	*p* ^c^	Pearson Correlation	*p* ^d^
Genotype								
TT	213.23 ± 258.53				4.44 ± 2.43			
CT	725.33 ± 998.49	0.028 *	0.25	0.036 *	4.43 ± 2.32	0.950	−0.001	0.997
CC	591.51 ± 999.63	0.093	0.16	0.116	4.83 ± 2.26	0.779	0.06	0.679
Dominant								
TT	213.23 ± 258.53				4.44 ± 2.43			
CC + CT	644.19 ± 997.37	0.044 *	0.15	0.071	4.62 ± 2.29	0.918	0.02	0.843
Recessive								
TT + CT	589.77 ± 893.25				4.43 ± 2.31			
CC	591.51 ± 999.63	0.982	0.001	0.991	4.83 ± 2.26	0.187	0.085	0.403
Overdominant								
TT + CC	519.84 ± 917.72				4.77 ± 2.26			
CT	725.33 ± 998.49	0.157	0.103	0.216	4.43 ± 2.32	0.209	−0.07	0.467

aCCP: anti-cyclic citrullinated peptide; RA: rheumatoid arthritis; SD: standard deviation; aCCP levels were expressed as mean ± SD; *p* ^a^: significance of differences in aCCP levels in patients with RA; *p* ^b^: significance of Pearson correlation in genetic subgroups and aCCP levels in patients with RA; *p* ^c^: significance of differences in aCCP levels in healthy controls; *p* ^d^: significance of Pearson correlation in genetic subgroups and aCCP levels in healthy controls. * *p* < 0.05.

**Table 5:** Regulatory motifs altered at rs878081, rs1003854, and rs1055311.

Locus	Ref	Alt	Position Weight Matrix ID	Strand	LOD Value of Ref	LOD Value of Alt
rs878081	C	T	NF-kappaB_known4	+	12.5	14.5
rs1003854	T	C	GATA_disc3	+	12.2	13.2
rs1055311	C	T	NF-kappaB_disc2	+	1.7	12.8

Ref: Reference allele; Alt: Alternative allele; LOD: logarithm of the odds; Available online: https://pubs.broadinstitute.org/mammals/haploreg/haploreg.php (accessed on 25 March 2024) [47,48].

## Data Availability

The data presented in this study are available upon request from the corresponding authors.

## Supplementary Materials

The following supporting information can be downloaded at: https://www.mdpi.com/article/10.3390/biology13060439/s1, Figure S1: Result plot of allelic discrimination test of rs878081 among RA patients. Figure S2: Result plot of allelic discrimination test of rs878081 among control subjects. Table S1: Allele and genotype frequencies, association of RA with allelic polymorphism rs2075876 in *AIRE*. Table S2: Allele and genotype frequencies, the association of RA with allelic polymorphism rs1055311 in *AIRE*.
