# Supplementary material for: Genetic Polymorphisms in Exon 5 and Intron 5 and 7 of AIRE Are Associated with Rheumatoid Arthritis Risk in a Hungarian Population
Source: Biology (Basel). 2024 Jun 15;13(6):439. doi: 10.3390/biology13060439 (PMC11200628; doi:10.3390/biology13060439)
Supplement: Supplementary file 1 [file biology-13-00439-s001.zip › biology-3031335-supplementary.pdf]

Supplementary Information

Genetic polymorphisms in Exon 5, Intron 5 and 7 of AIRE are associated with rheumatoid arthritis risk in a Hungarian population

Bálint Bérczi, Nóra Nusser, Iván Péter, Balázs Németh, Ágota Kulisch, Zsuzsanna Kiss and Zoltán Gyöngyi

Index

1. **Supplementary Figures**

   1.1. Supplementary Figure S1. Results of allelic discrimination test of rs878081 among RA patients

   1.2. Supplementary Figure S2. Results of allelic discrimination test of rs878081 among control subjects

2. **Supplementary Tables**

   2.1. Supplementary Table S1. Association of RA with allelic polymorphism rs2075876 in *AIRE*

   2.2. Supplementary Table S2. Association of RA with allelic polymorphism rs1055311 in *AIRE*

1. Supplementary Figures

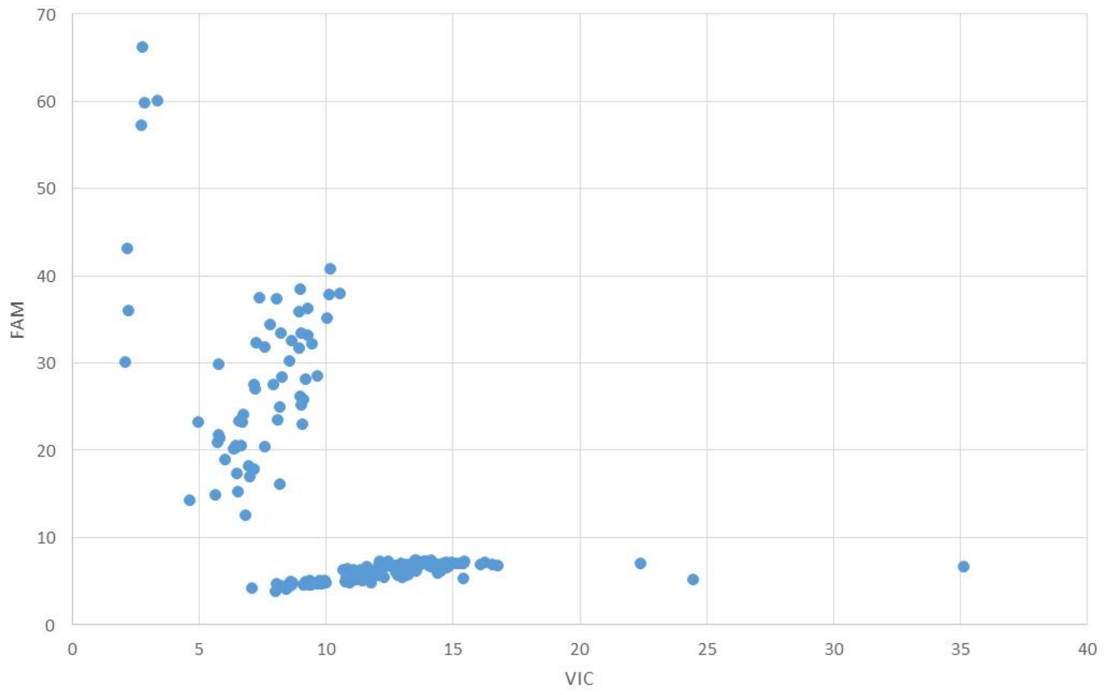

1.1. Supplementary Figure S1. Result plot of allelic discrimination test of rs878081 among RA patients. Signals only in the VIC™ channel represent homozygosity for C-allele and in FAM™ channel homozygosity for T-allele. Signals in both channels are considered heterozygous for rs878081 among RA patients.

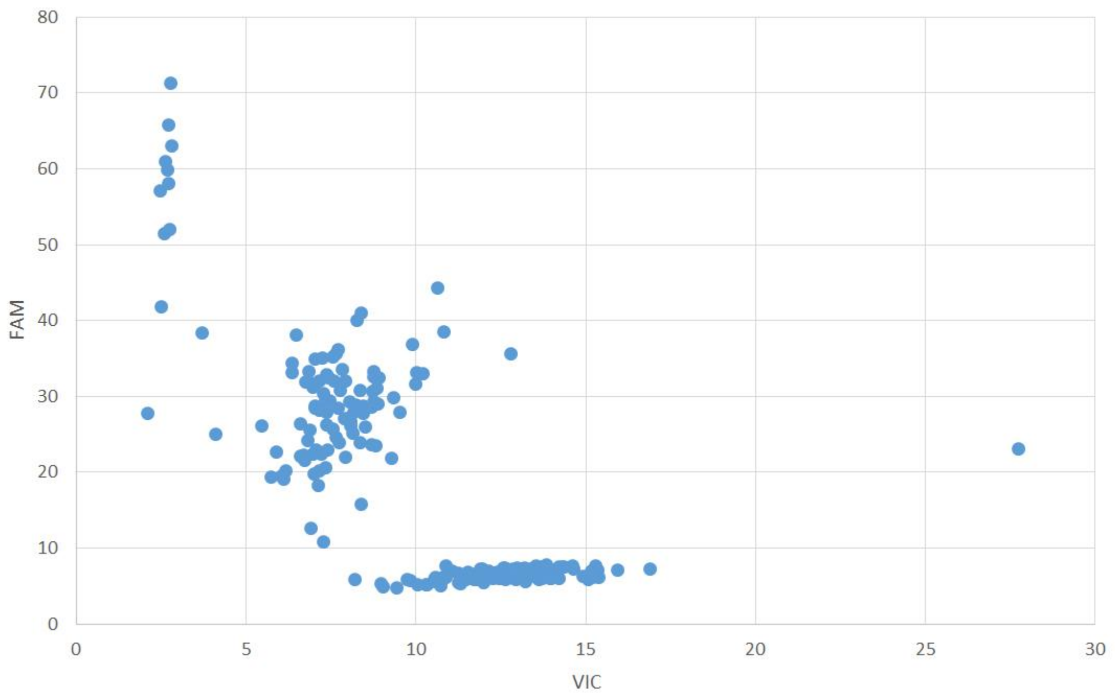

1.2. Supplementary Figure S2. Result plot of allelic discrimination test of rs878081 among control subjects. Signals only in the VIC™ channel represent homozygosity for C-allele and in FAM™ channel, homozygosity for T-allele. Signals in both channels are considered heterozygous for rs878081 among control subjects.

2. Supplementary Tables

2.1. Supplementary Table S1. Allele and genotype frequencies, association of RA with allelic polymorphism rs2075876 in *AIRE*.

AIRE rs2075876 (Intron 7)	RA N=270 (%)	Control subjects N=322 (%)	OR (95% CI)	p[a]
Alleles				
A	59 (10.9)	72 (11.2)		
G	481 (89.1)	572 (88.8)	1.02 (0.71-1.47)	0.890
Genotypes				
AA	3 (1.1)	8 (2.5)		
GA	49 (18.1)	54 (16.8)	2.42 (0.607-9.63)	0.210
GG	218 (80.7)	260 (80.7)	2.23 (0.58-8.53)	0.239
Dominant model				
AA	3 (1.1)	8 (2.5)		
GG+GA	267 (98.9)	314 (97.5)	2.26 (0.59-8.63)	0.230
Recessive model				
GA+AA	52 (19.3)	62 (19.3)		
GG	218 (80.7)	260 (80.7)	1.00 (0.66-1.507)	0.999
Overdominant model				
AA+GG	221 (81.9)	268 (83.2)		
GA	49 (18.1)	54 (16.8)	1.100 (0.71-1.86)	0.660

RA, rheumatoid arthritis; OR, odds ratio; CI, confidence interval; p[a]: significance of binary logistic regression.

2.2. Supplementary Table S2. Allele and genotype frequencies, association of RA with allelic polymorphism rs1055311 in *AIRE*.

AIRE rs1055311 (Exon 6)	RA N=270 (%)	Control subjects N=322 (%)	OR (95% CI)	p[a]
Alleles				
T	161 (29.8)	184 (28.6)		
C	397 (70.2)	460 (71.4)	0.94 (0.73-1.21)	0.639
Genotypes				
TT	25 (9.3)	24 (7.5)		
CT	111 (41.1)	135 (41.9)	0.798 (0.42-1.458)	0.450
CC	134 (49.6)	162 (50.3)	0.794 (0.43-1.454)	0.455
Dominant model				
TT	25 (9.3)	24 (7.5)		
CC+CT	245 (90.7)	298 (92.5)	0.78 (0.44-1.41)	0.428
Recessive model				
CT+TT	136 (50.4)	160 (49.7)		
CC	134 (49.6)	162 (50.3)	0.97 (0.704-1.34)	0.86
Overdominant model				
CC+TT	159 (58.9)	186 (57.8)		
CT	111 (41.1)	136 (42.2)	0.95 (0.68-1.32)	0.782

RA, rheumatoid arthritis; OR, odds ratio; CI, confidence interval; p[a]: significance of binary logistic regression.
